# Supplementary material for: Service access experiences of immigrant and refugee caregivers of autistic children in Canada: A scoping review
Source: PLoS One. 2023 Nov 9;18(11):e0293656. doi: 10.1371/journal.pone.0293656 (PMC10635503; doi:10.1371/journal.pone.0293656)
Supplement: S1 Appendix — (DOCX) [file pone.0293656.s001.docx]

Appendix A: Search Strands

The following are the search strands ran in each of the 4 databases utilized for this review. The dates at which these searches were ran by each independent reviewer has also been recorded.

**PsycINFO**

MAINSUBJECT.EXACT.EXPLODE("Autism Spectrum Disorders") OR noft(autism spectrum disorder*) OR noft(autis*) OR noft(asperger*) OR noft(ASD) OR noft(pervasive developmental disorder*) OR noft(PDD)

AND

MAINSUBJECT.EXACT.EXPLODE("Parents") OR MAINSUBJECT.EXACT.EXPLODE("Parenting") OR MAINSUBJECT.EXACT("Caregiving") OR MAINSUBJECT.EXACT("Caregivers") OR noft(mother*) OR noft(father*) OR noft(parent*) OR noft(caregiv*)

AND

MAINSUBJECT.EXACT.EXPLODE("Immigration") OR MAINSUBJECT.EXACT("Refugees") OR noft(immigrant*) OR noft(refugee*) OR noft(migrant*) OR noft(transient*)

*Ran August 10^th^, 2023*

**CINAHL w/ Full Text**

(MH "Child Development Disorders, Pervasive+") OR TI autis* OR AB autis* OR TI autism spectrum disorder* OR AB autism spectrum disorder* OR TI asperger* OR AB asperger* OR TI ASD OR AB ASD OR TI pervasive developmental disorder* OR AB pervasive developmental disorder* OR TI PDD OR AB PDD

AND

(MH "Parents+") OR (MH "Fathers+") OR (MH "Mothers+") OR (MH "Caregivers") OR (MH "Parenting") OR (MH "Parents of Children with Disabilities") OR TI parent* OR AB parent* OR TI father* OR AB father* OR TI mother* OR AB mother* OR TI caregiv* OR AB caregiv*

AND

(MH "Immigrants+") OR (MH "Emigration and Immigration") OR (MH "Transients and Migrants") OR (MH "Relocation") OR (MH "Refugees+") OR TI immigrant* OR AB immigrant* OR TI refugee* OR AB refugee* OR TI migrant* OR AB migrant* OR TI transient* OR AB transient*

*Ran August 21^st^, 2023*

**Medline**

MESH.EXACT.EXPLODE("Child Development Disorders, Pervasive") OR noft(autism spectrum disorder*) OR noft(autis*) OR noft(asperger*) OR noft(ASD) OR noft(pervasive developmental disorder*) OR noft(PDD)

AND

MESH.EXACT.EXPLODE("Parents:I.01.880.853.150.688") OR MESH.EXACT.EXPLODE("Parenting") OR MESH.EXACT.EXPLODE("Caregivers:M.01.085") OR noft(mother*) OR noft(father*) OR noft(parent*) OR noft(caregiv*)

AND

MESH.EXACT.EXPLODE("Emigrants and Immigrants") OR MESH.EXACT("Transients and Migrants") OR MESH.EXACT("Refugees") OR noft(immigrant*) OR noft(refugee*) OR noft(migrant*) OR noft(transient*)

*Ran August 21^st^, 2023*

**Sociological Abstracts**

MAINSUBJECT.EXACT.EXPLODE("Autism") OR noft(autism spectrum disorder*) OR noft(autis*) OR noft(asperger*) OR noft(ASD) OR noft(pervasive developmental disorder*) OR noft(PDD)

AND

MAINSUBJECT.EXACT.EXPLODE("Parents & parenting") OR MAINSUBJECT.EXACT.EXPLODE("Caregiving") OR MAINSUBJECT.EXACT.EXPLODE("Caregivers") OR noft(mother*) OR noft(father*) OR noft(parent*) OR noft(caregiv*)

AND

MAINSUBJECT.EXACT.EXPLODE("Immigrants") OR MAINSUBJECT.EXACT.EXPLODE("Refugees") OR MAINSUBJECT.EXACT.EXPLODE("Migrants") OR noft(immigrant*) OR noft(refugee*) OR noft(migrant*) OR noft(transient*)

*Ran August 21^st^, 2023*
