# Supplementary material for: Service access experiences of immigrant and refugee caregivers of autistic children in Canada: A scoping review
Source: PLoS One. 2023 Nov 9;18(11):e0293656. doi: 10.1371/journal.pone.0293656 (PMC10635503; doi:10.1371/journal.pone.0293656)
Supplement: Attachment — Submitted filename: Response to reviewers_LMC et al.docx [file pone.0293656.s002.docx]

Response to Reviewer Comments

To: Editors, PLOS ONE

Re: Manuscript ID PONE-D-22-26125

2023-October-13

Dear Editors,

Please accept this revision of our manuscript, “Service access experiences of immigrant and refugee caregivers of autistic children in Canada: A scoping review,” Manuscript ID PONE-D-22-26125. Please see below for our responses to each of the reviewer comments. We are also grateful for your accommodation of our delay in submitting this revision. The suggested changes took an extensive amount of time since they involved rerunning searches and analyses. We hope these revisions bring the manuscript up to an appropriate standard and are happy to make additional revisions as necessary. Thank you for providing feedback and suggestions to improve the manuscript, as well as for taking the time to review and consider this revised submission.

REVIEWER 1

Comment #1:

I would recommend the authors consider inspect the papers in some sort of chronological graph indicating linkages and themes (essentially a labeled flow-chart). This need not be a supplemental figure, but could be if it is productively illustrative. With these connections in hand, I would hope the authors could review their themes and perhaps observe whether some that have appeared in multiple papers did so independently - a mark of general importance above and beyond a mere “sticky” idea from an early paper, or if a less widely-cited theme has only recently cropped up (a possible mark of emerging importance).

Authors’ Response:

We revisited the themes identified in each study initially included in the review with the help of a non-author assistant. assistance of a research assistant. The assistant identified lines from the study that demonstrated key themes. The primary author then went through the sentences and highlighted specific words that captured the themes. The primary author then compared the similarities of the wording across studies and assessed the degree to which they were captured under the categories that were identified for the first draft of the manuscript. The authors felt that reviewing the original wording of the themes within the studies confirmed that the themes arose independently within the studies, and they were satisfied with how these categories captured those that arose in the studies. Moreover, the inclusion of the secondary review using an assistant helped to assess the level of bias in the original theme identification. New concepts from novel studies that were uncovered by modifying the searches as per Reviewer 2’s feedback were identified and added to the existing categories. The authors elected not to include the flowchart as a supplemental figure as the process did not lend itself to an illustrative chart that fit in cohesively with the rest of the paper; this process was instead described in the Methods section.

Comment #2:

The review summarizes demographic information from the cited papers (e.g., Figure 2), but could, in theory relate this information to broad immigrant / refugee demographics across Canada from other sources across the highlighted time frame. Given that the emerging consensus is that ASD prevalence (if not diagnosis) is uniform across ethnicities / countries of origin, a comparison of the published studies to broader demographics might highlight gaps in the space of the literature reviewed here. For a while in the United States there existed a lower rate of ASD in the latinx population, generally thought to be driven by a reduced rate of recognition/diagnosis. Without this first step of identification, these populations would be underrepresented in a review such as this. A comparison of general population demographics to those of the cited papers might cast light upon those populations currently overlooked in the literature.

Authors’ Response:

We procured relevant statistics on autism prevalence across immigrant and refugee groups to the best of our ability, as well as relevant statistics on the demographic breakdown of country of origin of immigrant and refugees in Canada, to the extent this data was available (see revised Figure 2). Where it was not available, the implications of this were acknowledged. We then included a written comparison of this data to the demographic data of the included studies in the Research Gaps section of the manuscript.

Comment #3:

I was left wondering about the demographic statistics of refugee families. My unfortunate suspicion is that a significant percentage of refugee families entering Canada are incomplete (e.g., missing a father). This seems like a potential for interaction with the larger theme of paternal involvement in caregiving with refugee status. This might be an informative point for discussion toward the particularities of the refugee experience and useful for future investigators.

Authors’ Response:

Attempts to further research the family composition of refugees in Canada were made and explored in the Research Gaps section of the manuscript, and the implications of the current limited availability of this data was discussed.

REVIEWER 2

Comment #1:

please include the vendor name for all databases.

Authors’ Response:

Vendor names for all the databases have been included in the Methods section of the manuscript.

Comment #2:

in the appendix please include the search strategies for all databases, copied and pasted exactly as run.

Authors’ Response:

Search strategies for all four of the databases included in this review have been copied into Appendix A.

Comment #3:

please use the most recent PRISMA flow diagram (2020). In the first box, please provide a breakdown of number of results per database.

Authors’ Response:

See Figure 1 in the manuscript for the PRISMA (2020) flow diagram, which now includes a breakdown of the number of results yielded from each database.

Comment #4:

as there were so few results, I would suggest that in your search, the phenomenon concept was unnecessarily focused and might result in missing relevant citations. I would omit that section of the search and screen for those concepts manually. A search for a scoping review should be broad to capture all available research.

Authors’ Response:

We revised the search strands with the assistance of a librarian. The phenomenon concept was removed from the search strands (see Appendix A).

Comment #5:

or population concepts 1 and 2 you have done a good job of using both subject headings and keywords. However, you did not do this in the other sections of your search. For population concepts 3a and b, please supplement the keywords with the appropriate subject headings. For example, exp Indians, North American/
Inuit/ exp "Emigrants and Immigrants"/ Refugees/ "Transients and Migrants"/ . Similarly for population concept 4, there is a subject heading for Canada: exp Canada/

Authors’ Response:

We revised the search strands with the assistance of a librarian to ensure the inclusion of appropriate subject headings across all search strands (see Appendix A).

Comment #6:

For the dates searched, please provide day and month of searches, as database results vary sometimes daily. Searches should be less than a year old. Your searches are a couple of years old. Please update your searches.

Authors’ Response:

Searches have been re-run and updated over the course of July-September 2023. Dates have been included in the Methods section of the manuscript.

Comment #7:

For screening of titles/abstracts, how many screeners did you have? There should be two independent reviewers. Similarly for data extraction, this should be done by two independent extractors.

Authors’ Response:

Two independent reviewers completed the screening of the included studies when the searches were rerun. We did not have the time and resources to go back and complete extraction independently, although we ensured that a non-author assistant worked with the primary author to re-examine the themes identified across the studies, as per Reviewer one's comments, to be as transparent as possible and to limit bias where possible. While screening in duplicate is common practice, and we have since completed this, duplicate extraction is not universally described as a necessary step in the published scoping review guidance we are aware of. We have clarified that data extraction was completed by only one reviewer in the Methods section.
